# Youth perceptions of how neighborhood physical environment and peers affect physical activity: a focus group study

**DOI:** 10.1186/s12966-015-0246-9

**Published:** 2015-06-20

**Authors:** Alan L. Smith, Philip J. Troped, Meghan H. McDonough, J. D. DeFreese

**Affiliations:** Department of Kinesiology, Michigan State University, 308 W. Circle Drive, East Lansing, MI 48824 USA; Department of Exercise and Health Sciences, University of Massachusetts Boston, 100 Morrissey Boulevard, Boston, MA 02125 USA; Department of Health & Kinesiology, Purdue University, 800 W. Stadium Ave, West Lafayette, IN 47907 USA; Department of Exercise and Sport Science, University of North Carolina at Chapel Hill, 209 Fetzer Hall, Chapel Hill, NC 27599 USA

**Keywords:** Built environment, Early adolescence, Peer relationships, Physical activity behavior

## Abstract

**Objective:**

There is need for a youth-informed conceptualization of how environmental and social neighborhood contexts influence physical activity. We assessed youths’ perceptions of their neighborhood physical and peer environments as affecting physical activity.

**Methods:**

Thirty-three students (20 girls; ages 12-14 years) participated in focus groups about the physical environment and peers within their neighborhoods, and their understanding of how they affect physical activity.

**Results:**

Inductive analysis identified themes of access (e.g., to equipment); aesthetics; physical and social safety; peer proximity and behavior (e.g., bullying); adult support or interference; and adult boundary setting. Participants also identified interconnections among themes, such as traffic shaping parent boundary setting and, in turn, access to physical spaces and peers.

**Conclusions:**

Young adolescents view neighborhoods in ways similar to and different from adults. Examining physical and social environments in tandem, while mindful of how adults shape and youth perceive these environments, may enhance understanding of youth physical activity behavior.

The structure of the physical environment that is deliberately constructed or modified by human activity, such as buildings, streets, and park or play spaces, is referred to as the built environment [1]. Physical properties of the environment have potential to affect physical activity, a behavior tied to important markers of physical and psychological health in young people [2]. Young adolescents spend substantial time in their neighborhoods because adults limit where they can go, they do not drive, and they lack financial resources. Accordingly, neighborhood features such as the presence of sidewalks, street connectivity, traffic, aesthetics and proximity to parks, schools, and places of business have attracted research attention.

Reviews of the literature on neighborhood built environment and youth physical activity show varying findings [3–5]. The most consistent environmental correlates of physical activity for children are traffic speed and volume, access and proximity to recreation facilities, walkability, mixed land use, and residential density, with the latter two most consistently supported for adolescents. Inconsistent evidence is found for parks and street connectivity, as well as recreation facilities for adolescents only [3]. Though adult studies have shown street connectivity to be positively related to physical activities such as walking [6], the importance of connected street patterns for youth activity is unclear [5, 7]. This inconsistency could reflect different functions of streets for adults versus children, as cul-de-sacs or low traffic areas that are less conducive to utilitarian walking for adults can afford safe locations for children to play. Inconsistent findings also can reflect use of varying measures of environmental attributes and physical activity [3]. It is evident that attention must be paid to the conceptualization and measurement of environment in this research area.

Often conceptual models and measurement approaches used with adults have been applied to studies of children and adolescents without sufficient consideration of developmental issues and young people’s perspectives. Cognitive, social, and biological differences between young people and adults can undermine the transferability of concepts and measures (see [8]). Young people likely have different perspectives on why they move through their neighborhoods (e.g., to find playmates versus obtain exercise) and factors constraining their movement (e.g., rules set by adults may be more salient than automobile traffic). They also likely perceive different affordances of features of their built environment. For example, an adult may view a garage door as helpful in securing and protecting valuable personal items, whereas a child may view the same door as a helpful backstop when shooting a basketball or playing tennis.

Developmentally informed work offers promise for advancing this research area. For example, recent work has employed an ecological momentary assessment methodology in generating understanding of where and with whom young people engage in leisure-time physical activity as well as the match of youth and adult perceptions of neighborhood environments [9, 10]. Participants carry a cell phone or similar device and respond to questions upon receiving random prompts. Questions about location and who someone is with can offer better understanding of use of the physical environment as well as social influences on physical activity. However, the questions are based on conceptions of the environment as it affects adult physical activity. Incorporating youth-based conceptions of physical activity environments is needed. Some physical environment surveys have incorporated young people’s perspectives (e.g., [11–13]). However, typically such efforts involve modifying adult measures, prioritizing parent over youth input, and basing physical environment conceptions on the adult literature. There is a clear need to understand what young people themselves view as important about their environments as related to physical activity.

Hume, Salmon, and Ball [14] had 10-year-old Australian children draw maps of their home and neighborhood environments and submit photographs of places and objects in these settings that were of personal importance. Opportunities for physical activity and sedentary behavior were among the themes that emerged from this activity, and some children included representations of yards, parks, or other green spaces. How frequently some of the qualitative elements appeared in the maps was associated with objectively measured physical activity behavior, though not entirely in expected directions. Opportunities for social interaction were also important to respondents. Overall, this work suggests that examining how young people perceive their physical and social environments as affording (or not) physical activity could benefit understanding of this important health behavior.

This type of research may be especially important for understanding physical activity behavior in adolescence. Early adolescents engage in more than half of their leisure time physical activity outdoors and away from the home, compared to less than one third for children ages 9 to 10 years [10]. This shift aligns with increasing independence from parents during early adolescence (see [15]). In considering barriers to active transport to parks, shops, and school, adolescents report higher concern with planning (i.e., logistical) and psychosocial factors than environment and safety, whereas parents report highest concern with environmental barriers [11]. This difference in adult and youth priorities suggests that it is important to consider youth perspectives regarding their own physical activity. Moreover, only 15 % of physical activity among youth is conducted alone [10]. This suggests that understanding neighborhood built environment and youth physical activity requires simultaneous consideration of the youth social context. This connection has been acknowledged in extant work [10–12] and aligns with ecological approaches emphasizing integrated, multi-level strategies for promoting physical activity (see [16]).

In the present investigation we targeted peers in the neighborhood environment. Peers are operationalized in a variety of ways, with emphasis typically placed on similarity in age, grade, or classroom, though also peers can be considered as those possessing similar skills, experiences, or backgrounds (see [17, 18]). Friends and broader peer groups can affect physical activity in young people through direct support, affiliation, modeling, victimization and other processes [17–19]. The degree to which built environment features are considered meaningful for physical activity by young people may tie to the degree that these features afford meaningful peer exchange.

There is need for a youth-informed conceptualization of how environmental and social neighborhood contexts influence physical activity. For early adolescents in particular, increasing independence from adults and salience of peers could result in perspectives unique from those of parents, other adults, and younger children. The purpose of our study was to develop such a conceptualization by assessing young adolescents’ perceptions of their neighborhood physical and peer environments as affecting physical activity.

## Methods

### Participants and neighborhoods

A convenience sample (*N* = 33; *n* = 20 girls) of seventh-grade students, ages 12 to 14 years (*M* = 12.5; *SD* = 0.62), was drawn from the public junior high-school serving a small city in the Midwestern United States. The population of the city and county was about 66,000 and 175,000 residents, respectively. Median annual family income was approximately $35,000 and about 65 % of students qualified for free or reduced price lunch (i.e., were from families with incomes at or below 130 % or 185 % of poverty level, respectively). Sixty–eight percent were involved in organized sport or physical activity and 15 % walked or bicycled to school. Participants were White (60 %), Black or African American (20 %), Hispanic (10 %), or Multiracial (10 %). Sample demographics were well aligned with the school population and sport participation was consistent with U.S.-based participation estimates by grade [20].

A Walk Score® and straight line distance in miles to the nearest school and park, respectively, were obtained for each participant’s home address (www.walkscore.com). Walk Score is a composite measure of neighborhood walkability based on walking routes to amenities, population density, and road metrics. Values range from 0 (low walkability/highly car dependent) to 100 (highly walkable/within a quarter mile of all amenities) and correlate with geographic information systems indicators of neighborhood walkability [21]. Participant residences had an average Walk Score of 40.1 (*SD* = 16.6; range = 8 to 72), indicating relatively car-dependent neighborhoods. Average straight line distance from home to school was 0.33 miles (*SD* = 0.38; range = 0.02 to 2.07 miles) and to the nearest park was 0.85 miles (*SD* = 0.53; range = 0.29 to 2.39 miles).

### Procedure

A youth-centered approach [22] was employed to understand and include young adolescents’ perspectives and voices and to expand theorizing and empirical knowledge in this area beyond adult perspectives. The philosophical position that social phenomena are individual and subjective, but have commonalities because they exist within social structures, was adopted [23]. Focus group methods were used to create opportunities for both individual responses and group interactions that allowed participants to hear, discuss, and make comparisons among each other’s opinions and experiences, potentially enriching the data [24].

Procedures were approved by the institutional review board at Purdue University and adhered to American Psychological Association ethical standards. Recruitment took place during physical education classes, which were compulsory for students in the district. The study purpose was described and study documents were distributed. Volunteers returned signed parent consent and participant assent documents. Focus groups were assembled based on the academic schedules, sex, and home address of participants. Seven same-sex focus groups consisting of three to six students were initially scheduled to be conducted. Same-sex focus groups were conducted in the interest of maximizing participant comfort to offer contributions to discussion. Participants living on the same street were assigned to different focus groups to enable, to the degree possible, heterogeneous neighborhood environments to be represented within a group. Focus groups followed Morgan’s [24] recommendations and lasted 30 to 40 min. Focus groups occurred during a school free or physical education period in a conference room. The third author, who possesses extensive experience in qualitative research methods, moderated the focus groups and the fourth author assisted. Focus groups were audio recorded. After the fifth focus group, the research team perceived that the themes communicated were redundant with those already documented. Therefore, additional focus groups beyond those initially scheduled were not expected to yield unique thematic information. The two remaining focus group interviews that were already scheduled were completed, confirming that expectation, and then data collection was discontinued.

The semi-structured focus group guide (available from the first author) was designed specifically for the present study, addressing the neighborhood physical environment and peers as tied to physical activity behavior. The guide was constructed by the lead author, reviewed by the research team, and then piloted with graduate-level students with expertise in developmental sport and exercise psychology. These students role-played youth participants during pilot execution of a focus group session, then suggested refinements to the guide and focus group delivery during a debriefing. The moderator first defined physical activity:

Physical activity is anything you do where your body is moving – walking, riding a bike, skate boarding, dancing, shooting hoops, playing catch with a friend, playing soccer on a team…any kind of physical movement you do. This includes any activities that make you breathe hard, make you sweat, or make your muscles tired; things like sports, skipping, running, climbing, and others.

Main questions addressed the typical activities of the participants, where they usually engage in the activities, what about those places makes them want to play or be active there (or not), and if neighborhood peers influence physical activity behavior. Follow-up probes addressed specific aspects of the neighborhood physical environment (e.g., size and proximity of structures, aesthetics, traffic, fences, working order of available equipment) and peer environment (e.g., number of same-age peers living nearby, how peers get together and if neighborhood physical layout influences this, if there are enough or too many children to play with). The assistant moderator maintained discussion notes and near the end of the session wrote a summary of the main themes on a white board visible to the group. The assistant moderator listed neighborhood physical environment and peer themes within two respective columns, verbalized the themes and asked if his understanding of the group discussion was accurate, and then asked participants about possible missing themes. Participants were then asked if they saw connections between the listed physical and peer environment themes. Following the discussion, participants were thanked and released as per school procedures.

### Data analysis

Audio recordings were transcribed verbatim and reviewed for accuracy. NVivo 8 (QSR International; Doncaster, Australia) software was used for data organization. Inductive content analysis [25] was used to analyze the data. Multiple researchers with different expertise and perspectives were involved in the analysis to account for multiple perspectives [26]. The first three authors independently reviewed all transcripts and inductively coded segments of raw interview data that corresponded to study questions. Coding involved inductively identifying segments that conveyed ideas relevant to the research question, and concisely labeling themes to convey the researchers’ interpretation of their meaning. These authors then met to discuss the segments and labels and to come to consensus, with the first author making final decisions on any disagreements. They then independently coded segments with common ideas into themes, and grouped themes addressing related ideas into common categories. Upon completing this inductive task, the authors met to compare coding and come to consensus on the content, labels, and groupings of themes and categories. As with initial coding of the raw data, the first author made final decisions when there were disagreements, which were very few. These authors then collectively identified and discussed interconnections of themes. The fourth author subsequently reviewed the findings and raised questions and offered suggestions for clarification. He compared the outcome of this process to his focus group discussion notes and in-session summaries of main themes to ensure the themes he perceived were represented. The overall process occurred over several weeks, enabling ample time for completion of the independent work and for reflection on group discussions and final interpretations. Quotations are labeled by the participant’s pseudonym, male (M) or female (F), and group number (three male and four female groups). The first appearance of a pseudonym is accompanied by the participant’s age in years.

## Results

### Themes

Six categories of themes were identified: access, neighborhood aesthetics, physical and social safety, peer proximity and behavior, adult support or interference, and adult boundary setting. *Access* pertains to the availability of age-appropriate equipment, street or yard space, or nearby community facilities linked to physical activity. One challenge raised by several of the groups was the age-appropriate nature of playground equipment at parks. Playgrounds were predominantly viewed as geared toward younger children and not meeting the needs of 12 to 14 year-olds: “At [the] park sometimes I feel like there needs to be stuff for, like, teenagers instead of little kids.” (Jenny, age 12, F/G2). Alternatively, sport-related equipment–most notably basketball hoops–and less constrained outdoor spaces were highly desirable if in good condition, not dominated by older youth or adults (see physical and social safety), and accessible by friends (see peer proximity and behavior): “I live right by my church…there’s a lot of basketball hoops and stuff like that. Also, there’s a big tree that you can climb in. So there’s a lot of things that you can do. A lot of my friends live there, so it’s a good place to go.” (Nicole, age 12, F/G4). Open spaces, particularly large yards, were highly valued: “We play…sometimes in the street or we just go find whoever has the biggest yard to play in.” (John, age 14, M/G3).

For some participants, fences offered direct opportunity for physical play, such as throwing or kicking a ball against a fence or as a boundary marker for a game. Others suggested that fences afforded safety and privacy to swim or play without concerns about being watched by others. At the same time, some participants recognized fences as deliberately placed barriers that would interfere with physically active play:

John – “Fences really concern me because…it sometimes shows that they want their privacy.” […]

Matt (age 13) – “…if there’s like no fences or no dogs outside, then I feel more welcome.” (M/G3)

Participants were likely to view yards as free-flowing spaces if fences were absent, suggesting they do not view property boundaries as salient unless deliberately marked: “I take shortcuts in between houses sometimes, like, if I just want to get home faster or something.” […] “But, like I know which shortcuts to take, so then I won’t like run into like a mad dog, or like, a fence or something.” (Heidi, age 12, F/G4).

*Neighborhood aesthetics* pertains to the appearance of the neighborhood that makes an area more or less attractive for physical activity. Participants valued a clean setting, absence of garbage and graffiti, and colorful and inviting equipment or space. Holes in or broken fences, presence of garbage, and presence of graffiti/vandalism made youth less interested in playing in a particular space, as did poorly maintained equipment:

Moderator – “Is there anything that makes a place somewhere you don’t want to go?” Ben (age 12) – “If it smells.” […] “Or if it has a lot of trash.” […]

Calvin (age 12) – “If it doesn’t have, like, good equipment, like, some of the equipment’s like rusted.” (M/G2)

However, examples were offered where negative features of a play space would be ignored when peers were available for play:

Heidi – “…the baseball diamond at [elementary school], it’s basically sand in like the shape [of a baseball infield] with a backstop. So that’s about it, but it works.”Moderator – “But you’d still go there?”Heidi – “Yeah, I mean it serves the purpose.” […]

Deanna (age 13) – “It really don’t matter what shape it’s in as long as you’ve got people to play with you and you can do it.” (F/G4)

Positive aesthetic features of the neighborhood made for an inviting physical activity setting, but negative aesthetic features were not a prominent barrier to activity when peers were available, providing these features did not signal a location as unsafe.

*Physical and social safety* was addressed in all of the focus groups. Concerns surrounding loose animals, vehicular traffic, and unsafe people make youth less comfortable doing physical activity. Loose animals were not of universal concern across the focus groups, though some participants noted concern about dogs: “…if there is just a big ol’ dog, I’m gone. If it’s…a big dog and it’s off the chain.” (Aaron, age 13, M/G1). The other safety themes were more commonly discussed. High traffic volume made an area less attractive, with youth more likely to seek large yard spaces or courts/cul-de-sacs, which have low traffic flow, for physical activities: “There is no traffic, so we can’t get bothered.” (Aaron, M/G1).

Linked with neighborhood aesthetics, graffiti cued participants to the possible presence of unsafe people in certain spaces. This included older teenagers, who were often viewed as using bad language and having poor judgment. Teenagers were considered intimidating:

Moderator – “Can you…elaborate a little bit? What would make someone scary?” Nicole – “Gothic.”

Heidi – “Um, people that are just kind of like skulking around, looking at you.”

Nicole – “Like if they have their hood up and just like.”

Deanna – “Like, if there’s like a bunch of people in a group.”

Nicole – “Yeah.”

Deanna – “With their hoods up and everything. That, that’s when I run.”

Nicole – “Punky. Punky.”

Moderator – “Okay, and again it’s sort of older kids or adults?

Nicole – “Yeah, it’s more like older kids, not as much as adults.”

Heidi – “Like, yeah like, fifteen to eighteen scare me the most.”

Nicole – “Yeah.”

Heidi – “It just seems like that’s like the most risky age.” (F/G4)

Presence of older teenagers and other unsafe people was particularly concerning in parks: “The only parks we have, like, they’re not very safe. Like there’s always older people, like, laying down on the benches and sitting there and watching you.” (Bree, age 12, F/G3). In one of the girls’ groups, sex offenders were noted to be of personal and parental concern:

Candice (age 12) – “My mom…makes us take cell phones [when going to the park] because she went online and saw…some sex offenders in our neighborhood.” Brittany (age 13) – “Yeah, my mom did that too.”

Alice (age 13) – “Yeah, my mom does that and we found out…one that lives like right around the block from us…” (F/G1)

*Peer proximity and behavior* included the presence of friendly, near-age peers (including siblings) who facilitated physical activity and the absence of peers who would discourage physical activity if they were present. It also included the absence of peers altogether, which for most participants deterred physical activity. As Rachel (age 13, F/G2) stated, “It’s sometimes hard [to play] because there’s barely anybody outside.” Being close in age was important because it increased likelihood of possessing common interests and increased perceptions of safety. Respondents wanted more same-age peers in the neighborhood; there never could be enough according to most. Bullying from older youth, however, could interfere with physical activity: “…a couple of kids that are about thirteen or fourteen. I have to put my bike inside my porch and lock it up because my last two bikes I had they used a hammer and broke [them].” (Tiffany, age 12, F/G3). Most participants believed that boys and girls could engage in physical activities together, but this was not uniformly accepted. For boys, it could depend on the specific girls: “If they’re like tough girls, and wouldn’t mind getting hit…” (Ben, M/G2), then boys would want to play with girls. Girls occasionally voiced concern about lack of common interests with boys, and bullying from boys that discouraged physical activity: “Some of the guys they like gang up on the girls and they just like make fun of them, like the way they look, and…they don’t want you playing with them and stuff. So then you just go inside and don’t have any fun.” (Brittany, F/G1). These barriers noted, having access to same-age peers with comparable interests was considered important for engaging in physical activity.

Though early adolescence is marked by increasing independence, adults were prominent in shaping young people’s neighborhood perceptions. Numerous examples of *adult support or interference* with physical activity were shared. Through interactions, rules, and expectations adults encouraged or discouraged physical activity. For example, parents in the neighborhood talked to one another about their children’s sport involvement or made equipment available, supporting physical activity. Alternatively, for some, the absence of involvement of particular adults made the neighborhood a preferred place to play: “There ain’t no coaches, so you ain’t gotta do no wind sprints, no, no running if you’re late. If you mess with people on the bus we ain’t gotta run 5 laps before our practice.” (Aaron, M/G1). More prominent, however, were concerns about neighbors who interfered with activity. Neighbors may voice concerns about noise, keeping their grass/yard nice, or potentially dangerous activities: “When I [throw a ball] against my fence, the neighbors usually [say] ‘Hey, stop that racket!’” (Calvin, M/G2). Tiffany (F/G3) noted: “I usually try to avoid this guy’s house because he’s like, he was an old farmer and he tries to keep his grass, like really, really perfect. And if I step on it he said he would tell the police on me…” Kelsey (age 13, F/G4) also shared how she no longer climbs trees: “I used to climb with my friend. But…it made it kinda hard ‘cause the lady didn’t like us doing that, even though it wasn’t her property. So, we can’t do that any more.”

In *adult boundary setting,* parents specify neighborhood limits and where and how their children can independently travel. Participants predominantly cited nearby parks/schools and busy roads as boundaries and noted that parents expect them not to cross those boundaries: “I have a busy street. And then sometimes my parents don’t want me going too far.” (Taylor, age 12, F/G3). Some participants mentioned other relatives’ houses as independent travel boundaries: “I don’t have a lot of places that I can go in between, in between my dad’s house and my grandma’s house.” (Matt, M/G3). Going to parks alone was discouraged for several of the girls: “I go to the park, and I have to go with my sister because my parents don’t think it is safe when I go by myself.” (Ashley, age 13, F/G1). From another girls’ focus group:

Jenny – “…usually I like, stay with my family. And I don’t go anywhere without any of my friends or anything.” […]

Rachael – “I wouldn’t go [to the park] within myself. But, I would go with someone else like, uh, one of my sisters.” (F/G2)

Parents also required their children to inform them where they were going or to carry a cell phone when traveling to the park or other locations not visible from the home: “I have to tell my parents where I’m gonna go.” (Calvin, M/G2).

### Interconnections among themes

When directly asked about connections between neighborhood physical and peer environment themes, participants found the query to be abstract and had difficulty conveying how these environments interface. This noted, discussions focused upon specific themes that revealed examples of interconnections between the neighborhood environment and peers. For example, participants avoided activities that require equipment for each person (riding bikes, playing on swings) if the amount of equipment was insufficient for the group: “The park that we go to it only has, like literally one swing that we can get in…when we come with our friends we always have to fight over the swing.” (Ashley, F/G1). Traffic and social safety issues were clearly tied to boundary setting by parents or other adults. This, in turn, had implications for access to physical spaces and peers that fall outside of those boundaries: “…I think there needs to be more kids my age, like right on my street because like all, bunch of the friends that I mentioned, they all live over in the other, like in the apartments. I can’t go get them and they don’t exactly come out all the time.” (Deanna, F/G4). Also, as Heidi (F/G4) expressed, “if you lived by something that you would need parental permission to like, go by, I’d think that there wouldn’t be as many kids.” Adult support for activity could come in the form of allowing access to yard spaces or providing a safe environment by watching out for young people. Such support offered opportunity to connect with peers and reduce parental safety concerns. Also, the presence of peers would draw participants outdoors, whereas the absence of peers would discourage going outside to play, even if attractive spaces and equipment were available: “If I can’t find anybody to play with usually I…go inside.” (Matt, M/G3). Finally, as noted previously, availability of peers made neighborhood aesthetics and equipment condition less important to physical activity choices.

## Discussion

This study examined how young adolescents perceive their neighborhood physical and peer environments as promoting or inhibiting physical activity. Several neighborhood environment features were tied to physical activity, including accessibility of spaces and equipment, aesthetics, safety, and peer proximity and behavior. Additionally, adults supported or interfered with neighborhood physical activity, as well as set boundaries. Though several themes (notably access and safety) are evident in adult-based work, early adolescent participants shared unique perceptions tied to their neighborhood context (e.g., issues pertaining to adult authority, activity affordances of objects such as fences and trees). Accommodating these perceptions in future youth physical activity research could help clarify mixed findings surrounding physical environment effects on physical activity.

Our findings align with work examining youth perceptions of the neighborhood. For example, in a sample of Canadian inner-city children of similar age, availability of physical activity resources (e.g., parks, playgrounds) and people-related safety concerns were key physical activity opportunities and barriers [27]. Notably, safety concerns restricted access to places to be physically active, making accompaniment by family members important to facilitating physical activity. As with the present sample, teenagers were viewed as engaging in risky or undesirable behaviors and as potential sources of bullying, a view shared by parents in other research [28].

In the present study safety concerns tied to use of physical activity resources such as parks stemmed from these spaces containing unknown people and often being out of the view of participants’ homes. Parks can possess many features that support youth being physically active, yet can be perceived as unsafe because they are unmonitored. This finding aligns with emerging work exploring adult perceptions of children’s free play and active transport that suggests fewer familiar adults available in the neighborhood during the daytime, reduced interaction among neighbors, and other demographic trends (e.g., smaller family sizes and greater mobility) contribute to elevated safety concerns [29, 30]. Accordingly, parents set boundaries and expectations that can reduce physical activity opportunities.

Adult boundary setting was tied to vehicular traffic and social safety. Some participants, particularly girls, were expected to travel with others for safety. However, they did not discuss boundaries pertaining to choice of playmates. This contrasts with Witten et al. [30], who found that parents commonly reported establishing rules pertaining to whom their 9 to 11 year-old children could affiliate. As young people enter early adolescence, it may become more difficult for parents to control a child’s social network. There could be value in future work that probes potential age-related trends in parental impact on both (1) the child social network and (2) where youth do and do not circulate within the physical environment.

Participants agreed that having more near-age playmates in the neighborhood was desirable. It appeared that there could never be enough peers, as this would afford greater opportunity to play. This aligns with findings from focus groups with 7 to 9 year-olds from suburban Western Canada, who indicated a willingness to play almost anywhere with anyone [31]. Availability of more peers is not only desirable to children and early adolescents, but could be viewed as a pathway to circumvent some parent concerns and boundaries. This, in turn, could offer greater access to outside play.

Young people appear more willing to tolerate less attractive surroundings and equipment when peers are available to them, speaking to the motivational salience of peers to physical activity (see [18]). Glenn and colleagues [31] found friends and siblings to be preferred playmates. Building upon friendship networks in the neighborhood would seem to afford physical activity opportunities that extend beyond the confines of the home, particularly if the networks consist of physically active friends (see [32]). With safety in numbers, adults may ease the boundaries they set, and youth may feel more comfortable outdoors and in less aesthetically pleasing surroundings. Examining how young people weigh aesthetic and social factors in their physical activity decision-making would be a valuable future research direction.

Our finding that ties exist between the neighborhood physical environment and peer context speaks to the efficacy of examining physical activity through an ecological systems theoretical lens [33, 34]. This conceptual framework has been strongly advocated in physical activity research [35, 36] and applied in extant work on young people’s physical activity contexts (e.g., [27, 28]). We argue that the particular value of this approach is in its capacity to direct attention toward the ties between various components and levels of influence on physical activity behavior. Understanding how environmental and social relationship components integrate to shape physical activity is critical to youth physical activity promotion.

A limitation of the present study is that it employs a convenience sample that represents one developmental group. Though several themes were similar to work with younger children and adults, there are developmental differences in youth perceptions of contexts for play and physical activity (e.g., [37]). Additionally, participants came from one community in a small Midwestern U.S. city. Perceptions may differ among youth in larger cities, rural communities, or different geographical locations in the U.S. and beyond. The school from which children were sampled had compulsory physical education, which is not universally adopted in schools. Young people’s perspectives on their physical activity contexts may be influenced by their exposure to educational messages and physical activity opportunities at school, as well as gender, ethnicity, race, socioeconomic status, and other factors not addressed in the present research. Expanding the samples employed in future work will enrich current knowledge. Such efforts could specifically recruit respondents who do not participate in organized sport or physical activities, or physical education, which would enrich understanding and draw from participants who might normally be less inclined to participate in research on physical activity behavior. Moreover, future research on the built environment–peer interaction using methods such as direct observation, neighborhood audits, objective assessment of physical activity behavior, and case studies would complement the present findings. Of particular interest would be observational work that speaks to the degree to which young people make adaptations to their environments (e.g., find ways to use playgrounds designed for younger children in ways that meet their needs) and if this flexibility/creativity is expressed in different ways developmentally. Finally, it was evident that adults remain significant social agents relative to the physical activity of young adolescents and likely impact the interconnection of physical and peer elements of the neighborhood environment. The interaction of parent and peer relationships is salient in youth activity settings [38], suggesting that exploring constellations of social relationships in the neighborhood will be important in future research.

Beyond demonstrating how the physical environment and peer availability interconnect as contributors to physical activity of early adolescents, the present study deepens our understanding through the voices of young people themselves. We encourage researchers to incorporate these voices into future studies that examine how neighborhoods influence health-related behaviors such as physical activity and that assess how the physical and social environments can inform the design of physical activity interventions for youth.
